# Short-Term Effect of Gabapentin on Subjective Tinnitus in Acoustic Trauma Patients

**Published:** 2017-03

**Authors:** Ali Goljanian Tabrizi, Abbas Safavi Naini, Nima Baradaran

**Affiliations:** 1 *Department of Otolaryngology, Shaheed Beheshti University of Medical Sciences, Tehran, Iran. *

**Keywords:** Acoustic trauma, Gabapentin, Subjective tinnitus

## Abstract

**Introduction:**

Although several treatment approaches have been proposed for tinnitus, there are currently no Food and Drug Administration (FDA)-approved agents available to treat this condition. In this study, we evaluated the effect of gabapentin on the sensation of subjective tinnitus in patients with acoustic trauma referring to the ear, nose and throat (ENT) clinic of Taleghani Hospital during 2014.

**Materials and Methods:**

In this double-blind, randomized clinical trial, 103 patients with tinnitus due to acoustic trauma who were referred to the ENT clinic of Taleghani Hospital during 2014 were randomized to the gabapentin (300 mg bid, n=55) or control (n=48) groups. The two groups were then compared before and after 6 weeks of treatment using a visual analog scale (VAS). At least a 30% reduction in VAS was considered a response to treatment.

**Results:**

Differences between the two groups regarding sex, age, duration of disease, and audiometry results was not significant (P>0.05). After 6 weeks’ treatment, the VAS significantly decreased in both groups (P<0.001), but the reduction was significantly greater in the gabapentin group compared with control (P<0.001). Forty-nine patients (89%) in the gabapentin group and 28 control patients (58.3%) responded to treatment (≥30% reduction in VAS), with the difference between the two groups being statistically significant (P<0.001).

**Conclusion:**

We conclude that gabapentin 300 mg bid for 6 weeks is an effective treatment for acoustic tinnitus. In addition, the placebo effect in relieving tinnitus is remarkable.

## Introduction

In tinnitus, the patient experiences abnormal auditory sensation without any evidence of an external acoustic stimulus. Tinnitus is classified into two types; objective and subjective. In the former, the noise heard has a physical explanation and is also perceived by the examiner, while in the latter type, the noise input *is *perceptible by the patient alone (1,2).

Subjective tinnitus is a relatively common disorder with a prevalence of approximately 32% in the United States of America (3). Several approaches for the treatment of tinnitus have been tried, including use of a tinnitus masker, biofeedback, electrical stimulation, hearing aids, surgical therapy, antidepressants, anxiolytics agents, and lidocaine, for example (4-8). However, these investigations are inconclusive and have shown limited success, with low patient satisfaction.

Because cerebral activity is altered in tinnitus, scientists have suggested alternative therapeutic approaches, such as anticonvulsant agents (9,10). The anticonvulsant agent, gabapentin, increases the synthesis of gamma-aminobutyric acid (GABA) in the brain and improves inhibitory neurotransmitter GABA activity. Moreover similar to lidocaine, gabapentin can inhibit voltage-activated Na^+^ channels (11). A study in rat models revealed that administration of salicylates decreases GABA and the affinity of the GABA receptors in the brain and induces the sensation of tinnitus and hearing loss (12). In keeping with this investigation, another study in rats reported that the reduction of GABA levels in the inferior colliculus induced hearing loss in these animals (13). Similarly, a further study on rat models documented that gabapentin significantly decreases tinnitus compared with placebo (14). Despite the positive results of gabapentin in the treatment of tinnitus, reports of the investigations are conflicting and most recommend further randomized clinical trials in order to evaluate the impact of gabapentin on tinnitus. Therefore, we conducted this double-blind, randomized clinical trial in order to compare the effect of gabapentin with placebo on the sensation of subjective tinnitus in patients with acoustic trauma.

## Materials and Methods

*Patients:*This double-blind, randomized clinical trial recruited 103 patients who were referred to the ear, nose, and throat (ENT) clinic of Taleghani Hospital in 2014 with subjective tinnitus after sound trauma. Patients were excluded if they met any of the listed exclusion criteria.

*Exclusion Criteria:*

 Exclusion criteria were as follows: acute or chronic otitis; history of diseases such as thyroid, rheumatoid or renal diseases; pregnancy; intake of drugs such as aspirin, aminoglycosides, or diuretics; use of chemotherapy agents such as cisplatin; age greater than 50 years and less than 18 years. In addition, patients presenting with tinnitus not due to sound trauma, such as vascular or psychiatric disorders or presbycusis, were excluded. A magnetic resonance imaging (MRI) scan and auditory brainstem response test were performed in all patients with neurologic symptoms or any central nervous system complication. The study was explained to all patients before enrolment, and signed informed written consent was taken. In addition, the study was approved by the ethics committee of Shahid Beheshti University of Medical Sciences.

*Study Design:*

 Patients were interviewed and demographic information such as age, sex, and employment status were recorded before auditory tests were performed in all patients. Patients were then randomized into two groups using sequential numbers such that the first number was given to the first patient who was then allocated gabapentin 300 mg/daily (gabapentin group, or case group, n=55). The next patient was given the next number sequentially, and allocated placebo therapy in the form of matching capsules (control group, n=48). The two groups were administered capsules twice a day. Both participants and study staff (site investigators and trial coordinating center staff) were blinded to treatment allocation.

Pure tone audiometry was used for auditory measurements, and the average thresholds at 500, 1000, and 2000 Hz was determined for assessment.

The severity of tinnitus was measured based on a visual analog scale (VAS) before treatment initiation and after 6 weeks of therapy. VAS is a psychometric evaluation of tinnitus severity consisting of 11 questions, in which the patient is asked to respond to each question on a scale from 0 to 10. Responses were recorded on a straight line on a graded scale, and the numerical charts were determined by the patient (15,16).

A reduction of more than 30% in the VAS was considered the minimum response to treatment. Patients were asked to refer to the ENT clinic in case of fever, drowsiness, or dizziness.

*Statistical Analyses*

Data were analyzed using SPSS Version 22. Categorical data are presented as numbers (%) and continuous data as mean ± standard deviation (SD). We used the Chi-square test to compare categorical variables and the Student’s t-test and paired t-test to compare continuous variables. p<0.05 was considered statistically significant.

## Results

This study evaluated 103 patients (55 male and 48 female) in two groups, with a mean age of 37.6 years and a mean duration of disease of 4.2 years. Most patients in each group were employed. Differences between the two groups regarding sex, age, duration of disease, and job were not significant. In addition, there were no significant differences in audiometry tests between the two groups (Table.1). 

**Table 1:** Patient characteristics and Audiometry test in the two study groups.

**Variable**		**Gabapentin**	**Control**	**P** ***-*** **value**
Age	Year	38.60±8.15	37.16±8.87	0.36
Sex	Male	44 (80%)	29 (60.4%)	0.02
	Female	11 (20%)	19 (39.6%)	
Job	Worker	28 (42%)	19 (40%)	0.18
	Employee	8 (14.4%)	16 (33%)	
	Army	11 (19.6%)	6 (12.5%)	
	Others	8 (14.4%)	7 (14.5%)	
Duration (year)	Year	4.76±6.85	3.35±2.24	0.19
Audiometry test	Right ear	10 (18.2%)	10 (18.2%)	0.19
	Left ear	13 (23.6%)	12 (25.0%)	
	Bilateral	32 (58.2%)	26 (54.2%)	

**Fig 1:**
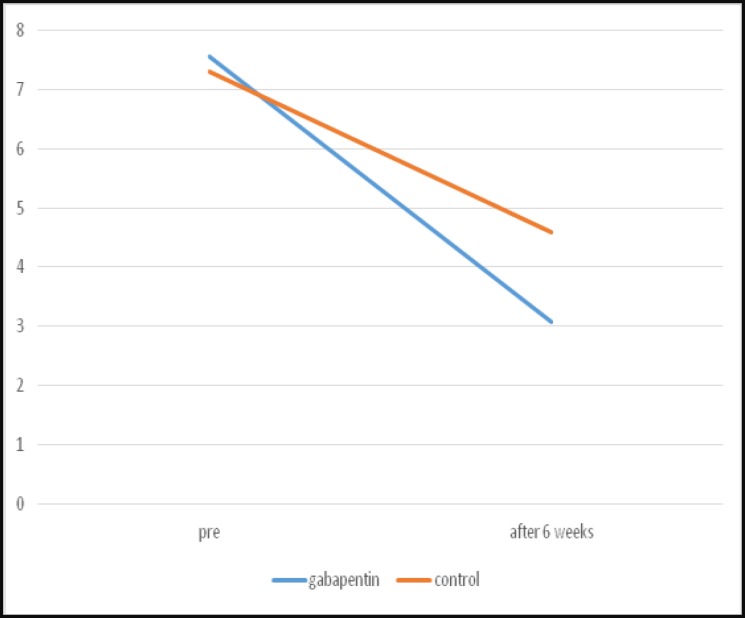
Severity of tinnitus based on a visual analog scale before and after 6 weeks of treatment in the two study groups

There was no significant difference in mean VAS between the two groups before treatment; however, after treatment the VAS score was significantly lower in the gabapentin group than the control group (Table.2, Fig.1). In addition, the difference between pre- and post-treatment in each group was significant. Furthermore, the number of patients achieving at least a 30% 

decrease in VAS (response to treatment) was significantly greater in the gabapentin group compared with the control group (Table.2).

 The correlation of response to gabapentin with age (P=0.83), disease duration (P=0.55), and sex (P=0.08) was not significant; however, response to gabapentin was significantly correlated with the employment status of the patient (P=0.01).

**Table 2:** Between-group differences in each group before and after treatment based on the visual analog scale

**Visual analogue scale**	**Before treatment**	**After treatment**	**95% CI**	**P** **-value (paired t-test)**
Gabapentin	7.56±1.01	3.07±1.11	4.10–4.87	<0.001
Control	7.31±1.01	4.60±1.25	2.28–3.12	<0.001
P-value (t-test)	0.21	<0.001		
≥30% decrease in VAS (response to treatment)	Gabapentin	Control	P-value (t-test)	
	49(89%)	28(58%)	<0.001	

## Discussion

Gabapentin has been used widely in several disorders, including seizure, anxiety, and pain. Researchers have demonstrated a high-affinity binding site in the synapses and have shown that gabapentin decreases the level of neurotransmitters by binding with calcium channel proteins and imitating the action of the inhibitory neurotransmitter GABA (17).

In the current randomized clinical trial, we compared the efficacy of gabapentin with placebo in patients with tinnitus referring to the ENT clinic because of acoustic trauma. The VAS and the Tinnitus Handicap Inventory (THI) are frequently used for the assessment of tinnitus. Although the THI is the most widely accepted method of tinnitus evaluation, the VAS tool is traditionally used, and there are a number of studies demonstrating a good correlation between VAS and THI scores. Thus, the VAS continues to be used as an appropriate method of measuring the intensity of *tinnitus* (18,19).

We found that gabapentin significantly decreased the VAS score in patients with tinnitus due to acoustic trauma. Moreover, gabapentin significantly increased the frequency of patients responding to treatment (≥30% decrease in VAS). Similar findings were reported in 2001 by Zapp et al. who indicated that gabapentin 500 mg daily increased the number of tinnitus-free days to 23 days/month (20). Similarly, in 2002 Shulman et al. revealed that the combination of gabapentin and clonazepam decreased the severity of tinnitus in 94.3% of patients with disabling tinnitus (21); a finding that was consistent with a 2001 investigation on psychophysical rat models (14). In addition, a study by Bauer and Brozoski indicated that gabapentin significantly decreased the loudness of tinnitus in adult patients who had a history of acoustic trauma (22). However, the results of treatment with gabapentin are not consistent, and some authors reported no significant effect of using gabapentin in tinnitus. For example, a 2011 controlled trial by Dehkordi et al. indicated that gabapentin had no positive effect on the treatment of tinnitus compared with placebo, although they reported that patients with hypertension, diabetes, and dyslipidemia all improved to a greater degree following treatment with gabapentin (23). Moreover, in double-blind clinical trials, Piccirillo et al. (24), Witsell et al. (25), and Bakhshaee et al. (26) concluded that the efficacy of gabapentin was not superior to placebo. Additionally, consistent with these findings, another study by Bahmad et al. investigating combination therapy with GABAergics and benzodiazepines in patients with severe tinnitus did not reveal any significant improvement among patients (27). The differences among these studies may be related to several factors such as differences in patient selection and methodology; moreover the definition of improvement may differ in these trials.

In the current study, the two groups were properly matched and differences in sex, age, and duration of disease were not significant between two groups, and thus had no effect on tinnitus response to gabapentin. However, the patients' employment status correlated with the results of treatment. Consistent with our investigation, Dehkordi et al. showed that a positive history of acoustic trauma, tinnitus sound type, accompanying symptoms, and duration of tinnitus had no impact on the outcome of treatment (23). Moreover, a review by Aazh et al. (28) was also in line with these findings, and did not reveal any correlation between duration of tinnitus and disease improvement. However, Aazh et al. indicated that the tinnitus duration may impact on treatment outcomes and patients, with a longer tinnitus duration leading to less improvement after treatment with gabapentin (28). A further study, by Otsuka et al., revealed that tinnitus patients aged more than 60 years showed a better response to intravenous lidocaine (29).

In summary, in the present trial we treated a case group of tinnitus patients with gabapentin 300 mg bid and found this to be an effective treatment. However, previous practices have used gabapentin at higher doses (600–1800 mg bid); therefore we recommend that gabapentin is initiated at minimum doses and increased if required. Although this study demonstrates strong evidence in favor of tinnitus treatment with gabapentin, some study limitations should be noted, such as the relatively small sample size and short duration of follow-up (6 weeks). These shortcomings may limit our ability to generalize the results of the present survey. Further analyses with larger series of patients and a longer duration of follow-up are required to determine whether gabapentin is an effective treatment for acoustic tinnitus.

## Conclusion

We conclude that 6 weeks’ treatment with gabapentin 300 mg bid is effective in patients with acoustic tinnitus. In addition, the placebo effect in decreasing tinnitus in these patients is remarkable.

## References

[1] Snow JB (2004). Tinnitus Theory and Management.

[2] Hall DA, Láinez MJ, Newman CW, Sanchez TG, Egler M, Tennigkeit F (2011). Treatment options for subjective tinnitus: Self reports from a sample of general practitioners and ENT physicians within Europe and the USA. BMC Health Serv Res.

[3] Mihail RC, Crowley JM, Walden BE, Fishburne J, Reinwall JE, Zajtchuk JT (1988). The tricyclic trimipramine in the treatment of subjective tinnitus. Ann Otol Rhinol Laryngol.

[4] House JW (1981). Management of the tinnitus patient. Ann Otol Rhinol Laryngol.

[5] Ireland CD, Wilson PH, Tonkin JP, Platt-Hepworth S (1985). An evaluation of relaxation training in the treatment of tinnitus. Behav Res Ther.

[6] Simpson JJ, Davies WE (1999). Recent advances in the pharmacological treatment of tinnitus. Trends Pharmacol Sci.

[7] Majumdar B, Mason SM, Gibbin KP (1983). An electrocochleographic study of the effects of lignocaine on patients with tinnitus. Clin Otolaryngol.

[8] Den Hartigh J, Hilders CG, Schoemaker JH, Hulshof JH, Cohen AF, Vermeij P (1993). Tinnitus suppression by intravenous lidocaine in relation to its plasma concentration. Clin Pharmacol Ther.

[9] Arnold W, Bartenstein P, Oestreicher E, Romer W, Schwaiger M (1996). Focal metabolic activation in the predominant left auditory cortex in patients suffering from tinnitus: A PET study with [18F] deoxyglucose. J Otorhinolaryngol Relat Spec.

[10] Lockwood AH, Salvi RJ, Coad ML, Towsley ML, Wack DS, Murphy BW (1998). The functional neuro anatomy of tinnitus: Evidence for limbic system links and neural plasticity. Neurology.

[11] Taylor CP, Gee NS, Su TZ, Kocsis JD, Welty DF, Brown JP (1998). A summary of mechanistic hypotheses of gabapentin pharmacology. Epilepsy Res.

[12] Bauer CA, Brozoski TJ, Holder TM, Caspary DM (2000). Effects of chronic salicylate on GABAergic activity in rat inferior colliculus. Hear Res.

[13] Milbrandt JC, Holder TM, Wilson MC, Salvi RJ, Caspary DM (2000). GAD levels and muscimol binding in rat inferior colliculus following acoustic trauma. Hear Res.

[14] Bauer CA, Brozoski TJ (2001). Assessing tinnitus and prospective tinnitus therapeutics using a psychophysical animal model. J Assoc Res Otolaryngol.

[15] Haefeli M, Elfering A (2006). Pain assessment. Eur Spine J.

[16] Meikle MB, Stewart BJ, Griest SE, Henry JA (2008). Tinnitus outcomes assessment. Trends Amplif.

[17] Taylor CP (2009). Mechanisms of analgesia by gabapentin and pregabalin-calcium channel alpha2-delta [cavalpha2-delta] ligands. J Pain.

[18] Figueiredo RR, Azevedo AA, Oliveira Pde M (2009). Correlation analysis of the visual-analogue scale and the Tinnitus Handicap Inventory in tinnitus patients. Braz J Otorhinolaryngol.

[19] Guimarães AC, Carvalho GM, Voltolini MM, Zappelini CE, Mezzalira R, Stoler G (2014). Study of the relationship between the degree of tinnitus annoyance and the presence of hyperacusis. Braz J Otorhinolaryngol.

[20] Zapp JJ (2001). Gabapentin for the treatment of tinnitus: A case report. Ear Nose and Throat J.

[21] Shulman A, Strashun AM, Goldstein BA (2002). GABAA-benzodiazepine-chloride receptor-targeted therapy for tinnitus control: Preliminary report. Int Tinnitus J.

[22] Bauer CA, Brozoski TJ (2006). Effect of gabapentin on the sensation and impact of tinnitus. Laryngoscope.

[23] Dehkordi MA, Abolbashari S, Taheri R, Einolghozati S (2011). Efficacy of gabapentin on subjective idiopathic tinnitus: a randomized, double-blind, placebo-controlled trial. Ear Nose Throat J.

[24] Piccirillo JF, Finnell J, Vlahiotis A, Chole RA, Spitznagel E JR (2007). Relief of idiopathic subjective tinnitus: Is gabapentin effective?. Arch Otolaryngol Head Neck Surg.

[25] Witsell DL, Hannley MT, Stinnet S, Tucci DL (2007). Treatment of tinnitus with gabapentin: A pilot study. Otol Neurotol.

[26] Bakhshaee M, Ghasemi M, Azarpazhooh M, Khadivi E, Rezaei S, Shakeri M (2008). Gabapentin effectiveness on the sensation of subjective idiopathic tinnitus: A pilot study. Eur Arch Otorhinolaryngol.

[27] Bahmad FM, Venosa AR, Oliveira CA (2006). Benzodiazepines and GABAergics in treating severe disabling tinnitus of predominantly cochlear origin. Int Tinnitus J.

[28] Aazh H, El Refaie A, Humphriss R (2011). Gabapentin for tinnitus: A systematic review. Am J Audiol.

[29] Otsuka K, Pulec JL, Suzuki M (2003). Assessment of intravenous lidocaine for the treatment of subjective tinnitus. Ear Nose and Throat J.

